# A Study of the Effects of Electrode Number and Decoding Algorithm on Online EEG-Based BCI Behavioral Performance

**DOI:** 10.3389/fnins.2018.00227

**Published:** 2018-04-06

**Authors:** Jianjun Meng, Bradley J. Edelman, Jaron Olsoe, Gabriel Jacobs, Shuying Zhang, Angeliki Beyko, Bin He

**Affiliations:** ^1^Department of Biomedical Engineering, Carnegie Mellon University, Pittsburgh, PA, United States; ^2^Department of Biomedical Engineering, University of Minnesota, Minneapolis, MN, United States; ^3^Institute for Engineering in Medicine, University of Minnesota, Minneapolis, MN, United States

**Keywords:** BCI, EEG, electrode number, CSP, channel configuration

## Abstract

Motor imagery–based brain–computer interface (BCI) using electroencephalography (EEG) has demonstrated promising applications by directly decoding users' movement related mental intention. The selection of control signals, e.g., the channel configuration and decoding algorithm, plays a vital role in the online performance and progressing of BCI control. While several offline analyses report the effect of these factors on BCI accuracy for a single session—performance increases asymptotically by increasing the number of channels, saturates, and then decreases—no online study, to the best of our knowledge, has yet been performed to compare for a single session or across training. The purpose of the current study is to assess, in a group of forty-five subjects, the effect of channel number and decoding method on the progression of BCI performance across multiple training sessions and the corresponding neurophysiological changes. The 45 subjects were divided into three groups using Laplacian Filtering (LAP/S) with nine channels, Common Spatial Pattern (CSP/L) with 40 channels and CSP (CSP/S) with nine channels for online decoding. At the first training session, subjects using CSP/L displayed no significant difference compared to CSP/S but a higher average BCI performance over those using LAP/S. Despite the average performance when using the LAP/S method was initially lower, but LAP/S displayed improvement over first three sessions, whereas the other two groups did not. Additionally, analysis of the recorded EEG during BCI control indicates that the LAP/S produces control signals that are more strongly correlated with the target location and a higher R-square value was shown at the fifth session. In the present study, we found that subjects' average online BCI performance using a large EEG montage does not show significantly better performance after the first session than a smaller montage comprised of a common subset of these electrodes. The LAP/S method with a small EEG montage allowed the subjects to improve their skills across sessions, but no improvement was shown for the CSP method.

## Introduction

Brain-computer interface (BCI) has attracted considerable attention during the past few decades and aims to construct a direct interface between the human brain and peripheral devices (He et al., 2013). Various signal sources such as endogenous motor imagery-based sensorimotor rhythms (Wolpaw and McFarland, 2004) and slow cortical potential (Birbaumer et al., 2003), and exogenous P300 (Jin et al., 2010, 2011) and steady-state visual evoked potentials (Chen et al., 2015) could be used to build the BCI system. Motor imagery-based BCI using electroencephalography (EEG) has shown promise in the control of virtual objects, such as computer cursors and virtual helicopters (Birbaumer et al., 2003; Wolpaw and McFarland, 2004; Royer et al., 2010), and physical devices, such as wheelchairs, quadcopters and robotic arms (Pfurtscheller et al., 2003; Carlson and Millan, 2013; LaFleur et al., 2013; Meng et al., 2016). Human brains display characteristic spatial modulation of sensorimotor rhythms when a user performs certain types of motor imagination, e.g., imagining single or bilateral hand movements, movement of the feet, etc. (Wolpaw et al., 2002; Pfurtscheller et al., 2006). This modulation of sensorimotor rhythms can be captured by computer algorithms and translated into various control commands for output devices. Recently, the study of non-invasive EEG-based BCI has sparked intensified interests, spreading to the inclusion of additional complimentary neurotechnologies such as EEG source imaging (Edelman et al., 2016), vivid imagination strategy (Qiu et al., 2017), hybrid modality (Kaiser et al., 2014), non-invasive neuromodulation (Baxter et al., 2016), and functional magnetic resonance imaging (Zich et al., 2015) to enhance the usability and performance of these systems. Additional studies have utilized EEG-based BCIs to explore neurophysiological foundations of BCI performance such as predicting user performance (Hammer et al., 2012), measuring brain plasticity through training (Pichiorri et al., 2011), the relationship between attempted movement and motor imagery (Blokland et al., 2015), as well as various clinical applications such as how BCI training may aid in stroke rehabilitation (Ramos-Murguialday et al., 2013; Pichiorri et al., 2015) and in the use of lower limb exoskeletons (King et al., 2015; Donati et al., 2016) and robotic arms for reach and grasp (Meng et al., 2016). These studies aim to further improve the performance of non-invasive BCI from various aspects in order to bring this technology into everyday life.

There are several factors, e.g., the chosen frequency bands, the channel configuration, the associated decoding methods, etc., which are known to affect the performance of BCI system (Blankertz et al., 2008b; Arvaneh et al., 2011; Ang et al., 2012; Meng et al., 2013). The selection of channel configuration, more often entangled with frequency/spectral optimization, and associated computer algorithms have been studied by various offline analyses (Lal et al., 2004; Blankertz et al., 2008b; Arvaneh et al., 2011; Meng et al., 2013). Despite a conflicting consensus within the field regarding the use of large numbers of EEG channels (electrodes) on BCI performance, it has been suggested by offline analyses that the classification accuracy of motor imagery/BCI tasks can increase as more channels are added (Lal et al., 2004; Sannelli et al., 2010; Arvaneh et al., 2011; Meng et al., 2013; Shan et al., 2015; Qiu et al., 2016), but begins to decrease after a certain number due to the redundant and/or irrelevant information introduced into the classifier. Usually, the optimal number of channels depends on the algorithm used, the subject and the application. One state-of-the-art signal processing method termed common spatial patterns (CSP) has gained significant attention due to its efficiency to extract useful motor imagery-related information from multiple channels (Blankertz et al., 2008b; Arvaneh et al., 2011; Meng et al., 2013). Several offline studies have shown that CSP works optimally when using about 20–50 electrodes, however, the optimal number varied among subjects and applications (Lal et al., 2004; Sannelli et al., 2010; Arvaneh et al., 2011; Meng et al., 2013; Shan et al., 2015; Qiu et al., 2016). Advanced signal processing techniques utilizing large numbers of EEG channels, such as CSP, may therefore additionally be able to boost BCI performance. Despite few online applications (Guger et al., 2000; Blankertz et al., 2008a), CSP has primarily been used to perform offline analysis of prerecorded EEG and has been compared to only one or two competing methods at a time (Guger et al., 2000; Blankertz et al., 2008a,b; Sannelli et al., 2010; Arvaneh et al., 2011; Lotte and Guan, 2011; Ang et al., 2012; Meng et al., 2013; Samek et al., 2014; Shan et al., 2015; Qiu et al., 2016). Furthermore, these studies often utilize data from one recording session and do not consider the learning process of subjects that may arise under longitudinal training paradigms. On the other hand, the LAP method uses a fixed configuration and a relatively small number of channels distributed around the sensorimotor area. There are multiple studies demonstrating online performance across several training sessions via the LAP method (Wolpaw and McFarland, 2004; Royer et al., 2010; Baxter et al., 2016; Meng et al., 2016), however, most of these studies do not investigate or report the learning process of subjects in part due to insufficient number of subjects and low statistical power. Meanwhile, many recent studies using BCI for stroke recovery, recovery of lower limb injury or robotic arm control highlight the requirement of online BCI learning in multiple/long-term sessions (Ramos-Murguialday et al., 2013; King et al., 2015; Pichiorri et al., 2015; Donati et al., 2016; Meng et al., 2016). Furthermore, the BCI learning of subjects in long-term sessions continuously occurs and interacts with the adaptation of the BCI system. Therefore, it is necessary to not only evaluate various channel configurations in an online fashion, but also to investigate how the use of these approaches affect the learning process involved in BCI control.

It is clear from many offline studies that the modulation of sensorimotor rhythms can be better captured by advanced signal processing algorithms (Guger et al., 2000; Lal et al., 2004; Blankertz et al., 2008a,b; Sannelli et al., 2010; Arvaneh et al., 2011; Lotte and Guan, 2011; Ang et al., 2012; Meng et al., 2013; Samek et al., 2014; Shan et al., 2015; Qiu et al., 2016) and may facilitate the learning process of BCI control. This facilitation might manifest in the form of higher decoding accuracies and overall BCI performance, which would possibly encourage the participants to maintain engagement throughout the training. However, to the best of our knowledge, it has yet to be tested whether or not the inclusion of large numbers of electrodes and advanced signal processing algorithms lead to more efficient BCI training and increased performance in an online setting. In the present study, we attempt to answer this question by investigating BCI learning over multiple sessions in terms of the performance of BCI control, a behavioral representation of the users' ability to modulate his/her sensorimotor rhythms.

In this study, we used two particular channel configurations, composed of vastly different numbers of electrodes in order to explore the learning performance of subjects. For each channel configuration, decoding algorithms were utilized that have been shown in literature to optimize the information collected among the included sensors; Laplacian Filtering (LAP/S) for the small channel configuration (Hjorth, 1975; McFarland et al., 1997), CSP/L for the large channel configuration, and CSP/S for the small channel configuration (Ramoser et al., 2000; Blankertz et al., 2008b). Forty-five participants were recruited to participate in this study. To address the question of how the channel configuration and decoding algorithm affects initial BCI learning, the subjects were randomly distributed into separate experimental groups utilizing different channel configurations and decoding algorithms. Each subject participated in multiple sessions of BCI experiments using a 1-dimensional (1D) cursor control paradigm. The group averaged performance across sessions was compared among the three groups.

## Methods

### Experimental setup

#### Subjects and data acquisition

**Figure 1:**
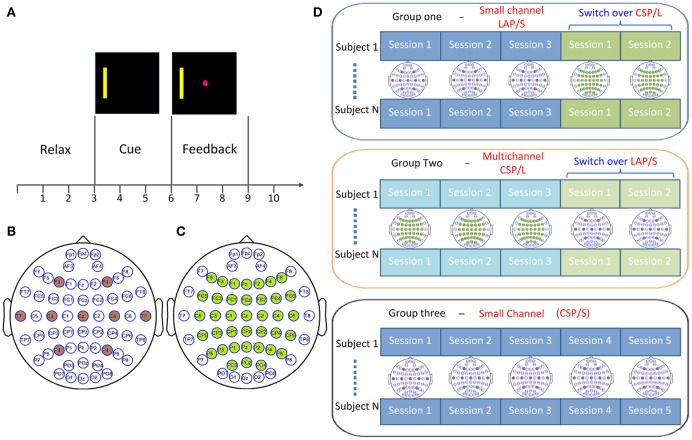
Experimental design. **(A)** Trial structure for cursor movement BCI control using left- and right-hand motor imagery. **(B)** Small channel configuration for large Laplacian filtering (LAP/S) method. **(C)** Multichannel configuration with 40 electrodes decoded by common spatial filter (CSP/L) method. **(D)** Switch-over design for the first two groups where participants were randomly distributed into two groups: each group performed three sessions of the original configuration and associated decoding method and then switched over to the other for the remaining two sessions; the channel configuration for each session is shown accordingly. Subjects in group three performed five sessions of online decoding by CSP/S but via the small channel configuration indicated in **(B)**.

Forty-five BCI naïve subjects were recruited for this study. The 45 subjects (22 females; mean age, 23.7 ± 7.7; range 18–54; seven of them are left handed, one ambidextrous) were randomly assigned to one of three groups **(Group one: G1, Group two: G2, and Group three: G3)**. For each subject, five sessions of 1D horizontal motor imagery BCI control (Figure 1A) were performed via either the small channel configuration (Figure 1B) or multichannel configuration (Figure 1C) and the associated online decoding algorithms. The average interval between any two of the five consecutive sessions for all subjects is 7.24 ± 8.85 days and the minimum interval for each subject is 1 day. The small channel configuration (**G1**, 15 subjects) employed the AR spectrum algorithm which extracted the amplitude of the alpha-beta rhythm (8–26 Hz) from channels C3 and C4. Before extracting the spectrum signals from those two channels, they were spatially filtered by LAP/S Filter (McFarland et al., 1997). Whereas, the CSP algorithm was used to adjust the weight coefficients applied to the multichannel configuration (**G2**, 15 subjects). Correspondingly, band-pass filtering (8–26 Hz) was employed to the multiple channels before the CSP algorithms was applied. As a control, the remaining 15 BCI naïve subjects **(G3)** were instructed to perform five sessions of the same BCI task via the small channel configuration and CSP decoding algorithm (CSP/S, same band-pass filtering). All participants were informed about the experimental protocol and written informed consent from all participants was acquired before participating in the study; the study protocol was approved by the Institutional Review Board (IRB) of the University of Minnesota.

64-channel EEG was acquired using a Neuroscan EEG acquisition system. The reference was located on the vertex and the ground electrode was on the forehead. During EEG recordings, subjects were seated in a comfortable chair with their hands on the armrests and faced a computer monitor at a distance of one meter. All electrode impedances were maintained below 5 kΩ. The EEG signals were recorded at a sampling rate of 1,000 Hz and band-pass filtered from 0.5 to 200 Hz by a Neuroscan Synamps RT amplifier (Neuroscan Inc, Charlotte, NC). A notch filter of 60 Hz was applied to the raw EEG signals.

### Study design

The 45 subjects who were naïve to BCI fell into one of the three groups randomly. Each subject was instructed to imagine movement of their left hand or right hand to control the left or right cursor movement, respectively. Subjects were instructed to perform kinesthetic motor imagination in the first person perspective (Neuper et al., 2005). There were 10 runs per session, each composed of 25 trials per run with left and right targets presented in block randomized order. Thus, the left and right targets were roughly equal in each session. In each session, there was no feedback for the first run of 25 trials and was used as the training data for CSP/L or CSP/S. There was no feedback for the first trial in each run as the data from this time period was used as training data (buffer initiation) for LAP/S. After the first training run or trial in the respective method, the feedback was provided to subjects. For each run, the trial started with a black screen for 3 s during which the subjects were instructed to relax and try their best to eliminate body movement. A yellow bar appeared after second 3 on either the left or right side of the screen and was maintained for 3 s, followed by the appearance of a pink cursor at second 6 which was allowed to move based on the subject's brain rhythms (as shown in Figure 1A). Subjects were given a maximum of 6 s in each trial to hit the correct target; thus, each trial could result in a hit, miss, or abort. After a 1 s post-feedback period a new trial repeated under the same procedure. The movement of the cursor was presented by BCI2000 (Schalk et al., 2004).

**Figure 2:**
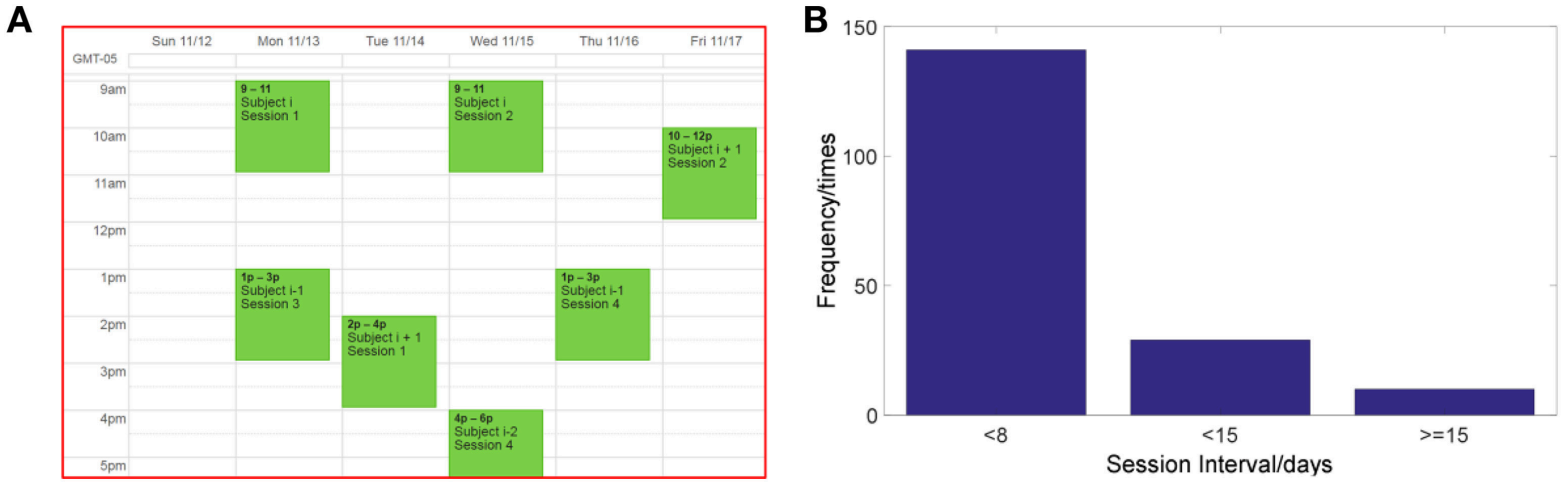
Schematic representation of the experimental schedule. **(A)** Scheme of subjects' recruitment and experimental schedule. **(B)** The statistics of inter-session interval for all sessions and subjects.

All of the subjects were blinded to which group they belonged to. The first group G1 included 15 subjects using the small channel configuration and LAP/S filtering method for online decoding during the first three sessions (G1:LAP/S); the last two sessions were switched to the multichannel configuration and CSP/L for online decoding (G1:LAP/S->CSP/L). For the second group, 15 subjects participated in three sessions of BCI control via the multichannel configuration and CSP/L as the online decoding method (G2:CSP/L) and then switched to the small channel configuration and LAP/S method for their last two sessions (G2:CSP/L->LAP/S, as shown in Figure 1D). The 15 subjects in the third group performed the same BCI task via the small channel configuration using CSP as the decoding method throughout the five sessions (CSP/S, G3:CSP/S). The ability to operate a BCI for participants is expected to be generalized from one decoding method to another if they are to use the same underlying neurophysiological mechanism. Changing the decoding method can act as a perturbation to a particular subject's control strategy, however, could also potentially help make such a control strategy more robust in the long run. The switch-over design among the first two groups was aimed to test how changing the decoding method would affect a subject's control strategy by means of behavioral performance. The experimental schedule for each subject was balanced between each subject's earliest next available day and our lab's availability; Figure 2A displays the scheme for recruiting and scheduling subjects. The statistics of inter-session interval for all subjects are displayed in Figure 2B.

### Multichannel (CSP/L) online decoding

Forty electrodes were selected and are marked green in Figure 1C. Channels at the periphery of the cap were ignored during online processing to avoid large, common artifacts caused by facial muscle movements. Those 40 channels were band-pass filtered by an 8–26 Hz Butterworth filter and then spatially filtered by three pairs of the most distinct CSP filters. The power in a sliding time window of 2 s in each of spatially and spectrally filtered EEG signals were used to calculate the movement of the cursor in each time step. CSP aims to maximize one class covariance while minimizing the other class covariance. The equivalent optimization problem aims to maximize one specific class covariance with the normalization constraint of two class covariance. The solution is given by the generalized eigenvalue decomposition (Ramoser et al., 2000; Blankertz et al., 2008b). Linear discriminant analysis (LDA) was used as the classifier. In the online BCI experiments in the current study, the normalized value of LDA output was mapped to the cursor velocity in either the positive (right) or negative (left) directions. For the first run of each session, the cursor did not move but the subject was required to do the same motor imagination as subsequent runs in order to get the training data to train the CSP and LDA classifier. After the first run, the spatial filters and LDA classifier were retrained based on the data of the previous two runs, 50 trials, (for the second run, only the data of first run were used). A batch mode (Qin et al., 2007; Meng et al., 2014) was applied to update the classifier online after each run.

### Small channel (LAP/S) online decoding

The signals of channel C3 and C4 were spatially filtered by LAP filter (Figure 1B) before they were used to calculate the power spectrum. Spectral power of the alpha-beta rhythm estimated by the Autoregressive (AR) method for channel C3 and C4 was input to a linear classifier and linearly mapped to the 1D left/right cursor velocity (LaFleur et al., 2013; Meng et al., 2016). For the first run of each session, the cursor did not move for the first trial but the subject was instructed to do the same motor imagination in order to calibrate a normalizer, after the first trial the cursor began to move (feedback). The normalizer took the output of the linear classifier as input and transformed it into a zero mean and unit variance control signal for velocity-based cursor control. The output of linear classifier was updated online by the normalizer (Schalk et al., 2004; Baxter et al., 2016).

### CSP/S online decoding

The signals from the small channel configuration (Figure 1B) were used for this paradigm and the same procedures of CSP and LDA were used to extract three pairs of features and classify the movement of the cursor. Similarly, the spatial filters and LDA classifier were updated online in a batch mode after each run.

### Group R-squared evaluation in sensor space

We used the coefficient of determination, R-squared (*r*^2^)value, to measure how strongly the means of the two distributions (left and right hand imagination) differ relative to the band power variance. In an offline analysis, trials contaminated by artifacts were removed to alleviate the obscurity of cortical activity caused by any electrical sources unrelated to the task, e.g., swallowing or head movement. Trials were rejected if the activity satisfied one of the following criteria: first, the power spectrum of the trial during the feedback period deviated from the baseline by ±35 dB in the 7–35 Hz frequency band (Delorme and Makeig, 2004); second, the feedback duration was <2 s. All of the trials including hit, miss and abort trials are used for the calculation of R-squared value. Artifactual trials were removed as described above in order to utilize only clean EEG signals, which could accurately capture the intention of the subjects. This procedure left an average of 200 ± 37 trials remaining for each subject and each session. A large Laplacian filter was applied to all of the recorded data (Hjorth, 1975; McFarland, 2015). Since a broad frequency band of 8–26 Hz was applied to all of the methods online, it is desirable to see the changes of R-squared values across sessions in the same frequency band for offline analysis. R-squared values were first calculated in each electrode in the frequency band of 8–26 Hz from all of the non-rejected trials for each subject and each session. Then the R-squared values were averaged over the subjects in each session.

## Results

### Online BCI performance results

**Figure 3:**
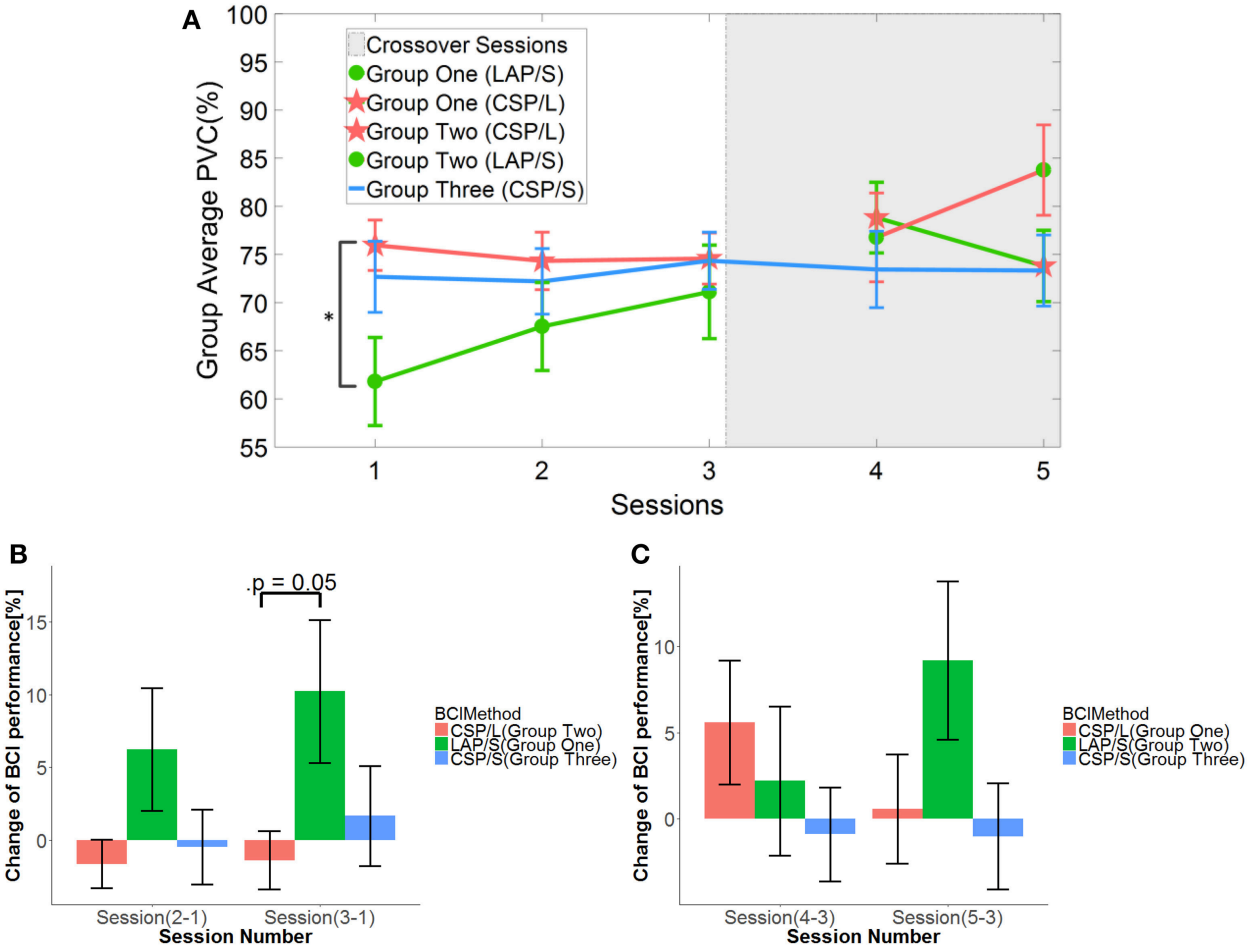
Online BCI performance and statistical analysis results. **(A)** Online group average performance in terms of Percent Valid Correct (PVC) across all five sessions. The standard errors of the mean (S.E.M) are indicated by the error bars on each point along the three lines. The same group of participants is connected by the same color lines and the same method is marked by red star and green circle, respectively. The “*” indicates there is a significant difference of performance between LAP/S and CSP/L at the first session. **(B)** Change of BCI performance across the first three sessions relative to the first (baseline) session. A significant difference between the group using CSP/L and the group using LAP/S is apparent in the overall improvement of PVC from the first to third BCI session. **(C)** Change of BCI performance compared to the third (baseline) session after switching over methods.

First, the online BCI performance for each method was averaged over groups of subjects for each session. The group averaged percent valid correct (PVC) for all of subjects in different groups across the five sessions is shown in Figure 3. The PVC is defined as the ratio of the correct target hit trials vs. all of the valid outcomes. Thus, invalid outcomes corresponding to those trials when neither a correct nor an incorrect target was hit (abort) were excluded in the calculation of PVC. The first group is indicated by the green line, the second group the red line, and the third group the blue line. Because of the switch-over employed in sessions four and five for the first two groups, the line color remains the same for original groups, however, the markers switch. Thus, a green line with green circles represents the first group for the first three sessions, whereas a green line with red stars represents the same group in the final two switch-over sessions. The same visualization method is applied to the second group (refer to Figure 1D for details). The light-gray highlighted region indicates when the switch-over sessions began.

A linear mixed effect model (lme) was employed to evaluate the statistical significance of group performance over time (across sessions) with *post-hoc* Tukey's tests used to correct for multiple comparisons. There was a significant difference (*p* = 0.03) in PVC between G1 (LAP/S, PVC ± S.E.M was 61.8 ± 4.6%) and G2 (CSP/L, 76.0 ± 2.6%) and no significant difference between G1:LAP/S and G3 (CSP/S, 72.7 ± 3.7%), or G2:CSP/L and G3:CSP/S in the first session. There were no significant differences among any group pairings in the following sessions. In the fifth session, we found a significant difference in PVC between G2 (LAP/S, 83.8 ± 4.7%) and G1 (CSP/L, 73.8 ± 3.7%), and between G2 (LAP/S, 83.8 ± 4.7%) and G3 (CSP/S, 73.3 ± 3.7%) before correction of multiple comparisons, however, these effects did not survive the correction. The group averaged PVC using the CSP decoding method and multichannel configuration (CSP/L, marked with red stars) showed significantly higher initial performance than the LAP/S decoding method using the small channel configuration at the first session. However, the performance of this group varied across the first three sessions without a clear trend and the variation (red star) continued after switching over to the LAP/S method as well. A similar high group average PVC by the small channel configuration (CSP/S, blue line) was shown at the first session, but no significant difference was shown compared to LAP/S decoding method. There was small variation in the performance for group three using the CSP/S method across the five sessions. The performance via the LAP/S decoding method using the small channel configuration (green circle) displayed a significant improvement, which is further discussed in section Offline BCI Performance Analysis, from the first to third sessions despite starting from a lower initial group averaged PVC. The change of BCI performance between session 4 and session 5 after switching over also shows greater improvement in group G2:CSP/L->LAP/S compared to G1:LAP/S->CSP/L (see analysis results in section Offline BCI Performance Analysis as well). In the following subsections, whether the randomly assigned subjects in three groups had equal natural abilities to control a BCI and whether the three methods produced different longitudinal effects of BCI control across the first three sessions are evaluated.

### Evaluation of BCI performance progress for different methods

**Figure 4:**
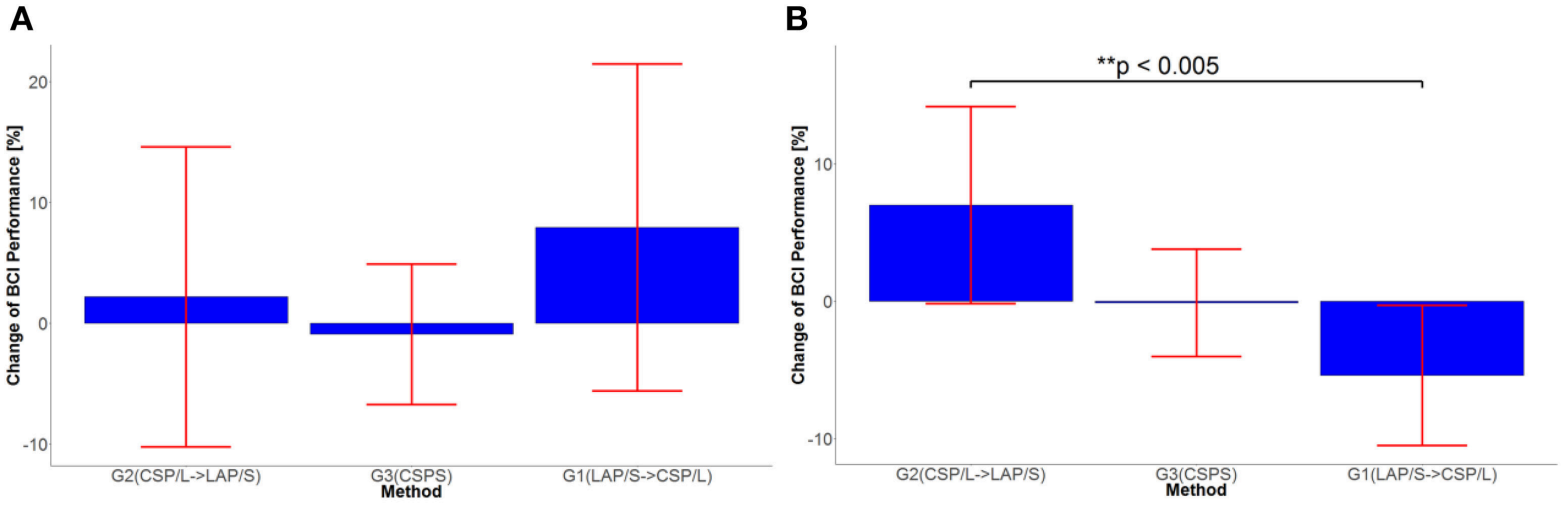
**(A)** Statistical significance test of change in performance between session 3 and session 4 for all three methods. **(B)** Statistical significance test of change in performance between session 4 and session 5 for all three methods.

In order to see the effects of the three different methods (here denoted as method A-LAP/S; method B-CSP/L and method C-CSP/S) and time (different sessions), the first three sessions and the last three sessions were analyzed separately. The performance of the first session (baseline) was subtracted from the second and third session to get the change of performance from baseline for each subject after training. Similarly, the performance of the third session was subtracted from the fourth and fifth to obtain the change of performance from the time point right before switching over methods for each subject. Although the offline cross validation analysis in section Offline BCI Performance Analysis showed that there was no significant difference in discriminative abilities between groups before the first online session (the offline cross validation in section Offline BCI Performance Analysis was used to assess subjects' discriminative abilities between groups), the difference in subject ability could be further compensated and evaluated by comparing their change of performance session by session. Thus, by baseline correcting the subjects' performance, the effect of initial group differences can be minimized. The change in performance (dependent variable, DV) for three different subgroups underwent treatment A, B, and C, respectively, for two different time points (repeated measures). A mixed repeated measures ANOVA was used to determine whether the three different methods produced different BCI performance over time. Prior to switching over methods, the statistics for the main effect of method is *F*_(2, 42)_ = 3.23, *p* = 0.049, *n*^2^ = 0.1 (generalized Eta-Squared measure of effect size); main effect of time is *F*_(1, 42)_ = 1.12, *p* = 0.30, *n*^2^ < 0.01; interaction effect of method and time is *F*_(2, 42)_ = 0.29, *p* = 0.75, *n*^2^ < 0.01. The statistics indicate there is a significant difference in the main effect of method. *Post-hoc* linear mixed effect models (lme) and Tukey's Test were performed between each method for multiple comparisons of means. The results are summarized in the Figure 3B. There is a marginally significant difference (*p* = 0.05) in change of BCI performance from session 1 to session 3 between method G1:LAP/S–G2:CSP/L (PVC ± S.E.M, 11.6 ± 5.0%); no significant difference (*p* = 0.81) between method G3:CSP/S–G2:CSP/L (3.0 ± 5.0%); no significant difference (*p* = 0.20) between method G3:CSP/S–G1:LAP/S (−8.6 ± 5.0%). There are no significant differences in the change of BCI performance from session 1 to session 2 (i.e., Session 2-1) among all of the three methods. After switching over methods, a similar mixed repeated measures ANOVA was used to assess the three different methods. No significant difference in main effects was shown; the statistics for the main effect of method is *F*_(2, 42)_ = 0.98, *p* = 0.39, *n*^2^ = 0.04; main effect of time is *F*_(1, 42)_ = 0.17, *p* = 0.68, *n*^2^ < 0.01. There was a significant interaction effect between method and time, *F*_(2, 42)_ = 5.35, *p* = 0.009, *n*^2^ = 0.03. Since a significant interaction was found, we did further *post-hoc* analysis on the change of BCI performance between session 4 and session 3, between session 5 and session 4 to find the reason for such significant interaction. The results are shown in Figure 4. A significant difference between G2: CSP/L->LAP/S and G1:LAP/S->CSP/L was shown for the change of BCI performance between session 4 and session 5 (see Figure 4B).

A linear mixed effect model was applied to test whether the carry-over effect was significant via the sequence of G1 (LAPS, session 3) (CSPL, session 4) and the sequence of G2 (CSPL, session3) (LAPS, session 4) in order to exclude the possibility of biasing the results for session 4 and session 5 due to the switch over design (Lawson, 2014). The result showed that there is no significant carry-over effect (*p* = 0.67) between the two sequences.

### Offline BCI performance analysis

**Figure 5:**
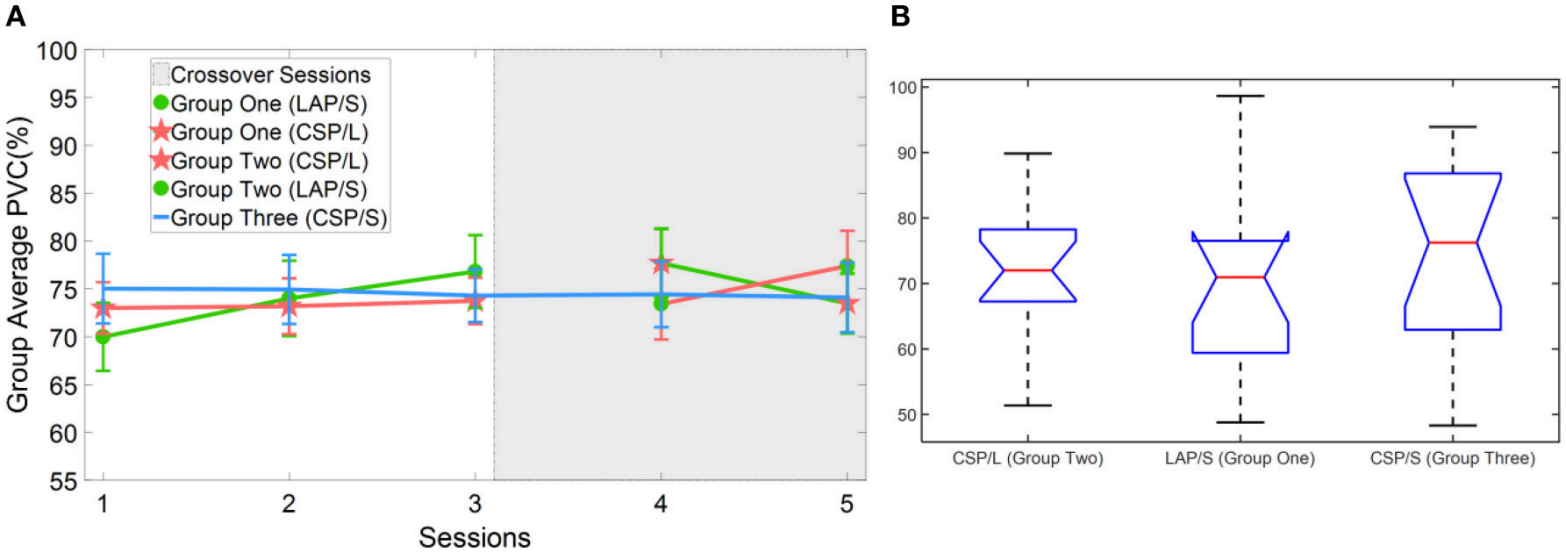
Offline cross-validation BCI performance and statistical analysis results. **(A)** Offline group average BCI performance via CSP/L method and a 5 × 5 fold CV. **(B)** One way ANOVA test on the groups' first session of offline BCI performance for three different groups. No significant difference was found.

Although the subjects were recruited and randomly assigned to different groups, it is worth investigating the genuine ability of each group to produce discriminable brain patterns and ensure that this ability was roughly equal. Then we can exclude the possibility that the difference of online performance between different groups, especially at the first training session, is caused by subjects' natural BCI ability. The offline analysis was performed for all of the subjects on their first session and subsequent four sessions of BCI data. Since CSP has been used as a benchmark method for numerous offline analyses (Blankertz et al., 2008b; Tangermann et al., 2012), CSP/L with the large channel configuration and a time segment of 2 s right after the cursor appears (3 s after the target cue was presented) was used as the method to assess the discriminability of subjects in all three groups. A 5 × 5 fold cross-validation (CV) was used to estimate the offline performance accuracy for each subject and each session. The group average CV accuracy in each group and session is shown in Figure 5A. Particularly, a one-way analysis of variance (ANOVA) was used to test whether there were statistically significant differences between the means of three groups at the first session. The average CV results and SEM for each method were G1:LAP/S (PVC ± S.E.M) 70.0 ± 3.6%, G2:CSP/L 73.0 ± 2.7% and G3:CSP/S 75.0 ± 3.7%. The statistical results of F(2,42) = 0.46, *p* = 0.63 indicate that there was no statistically significant differences between the means of the three groups' offline performance at the first session. Thus, we can conclude that the genuine ability to produce discriminable brain patterns in different groups was roughly equal.

### Group average R-squared value in sensor space

**Figure 6:**
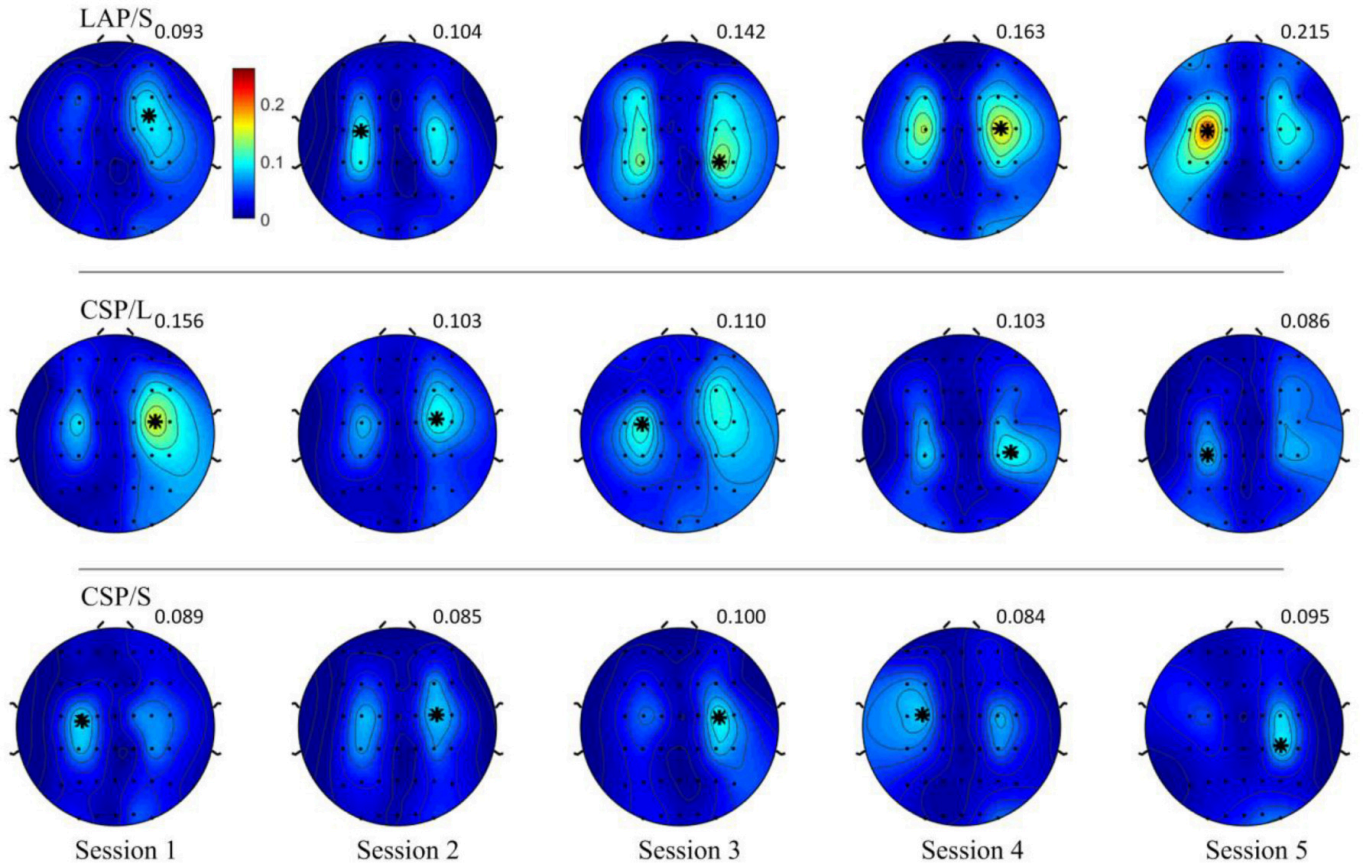
Topography of group averaged R-squared values for the three online decoding methods across five sessions. Each row shows the group results with respect to the different methods, and each column shows the results with respect to each session. The color scale for each method and session is globally normalized. The maximum R-squared value for each condition is marked as a black star and its value is shown rightly above each topography. The group averaged R-squared values for the LAP/S method displayed the largest R-squared value in the focal area surrounding channels C3 and C4 at the fifth session.

**Figure 7:**
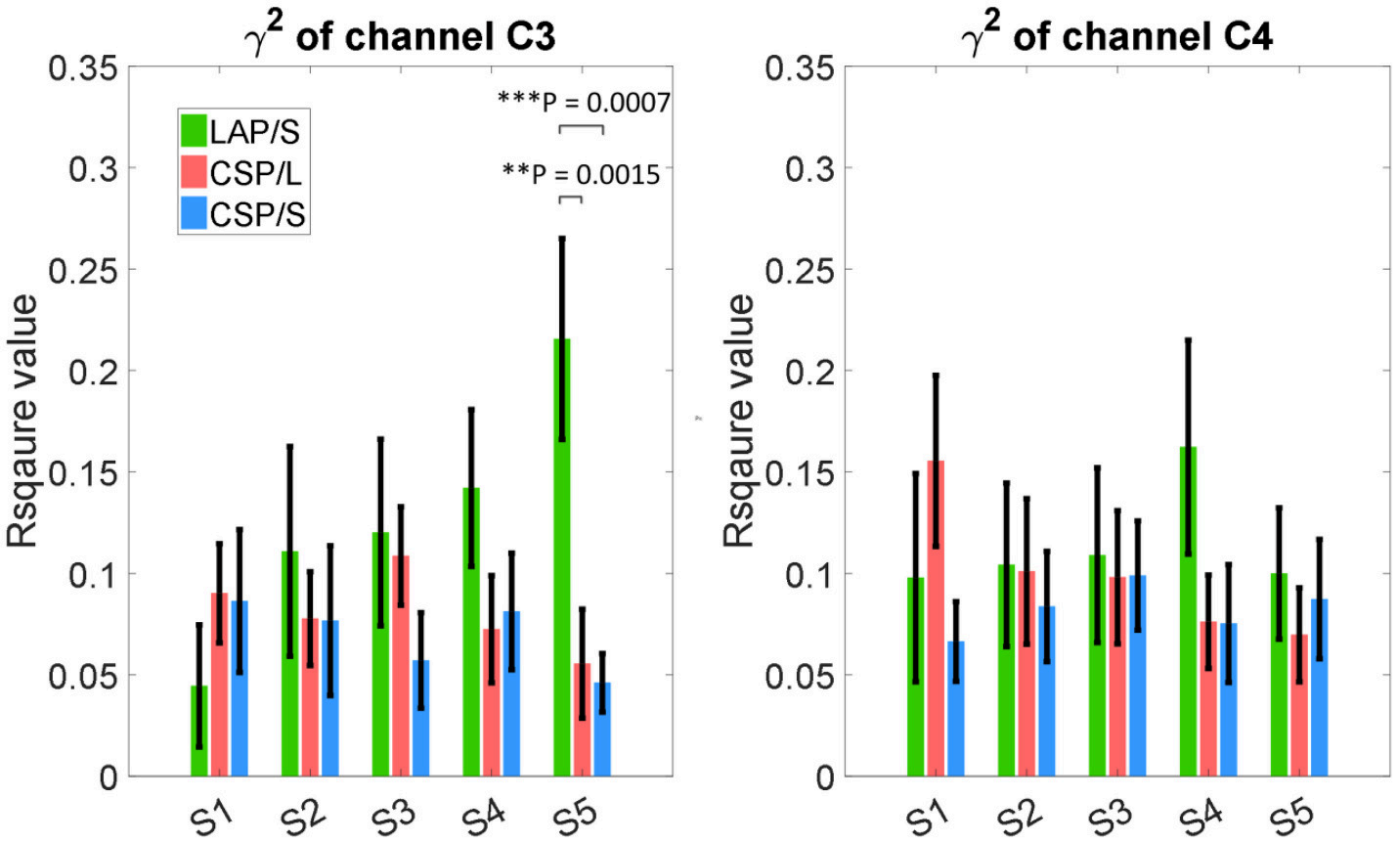
Statistical analysis of group averaged R-squared values, in channels C3 and C4, for the three online decoding methods across five sessions. Group mean ± S.E.M (standard errors of the mean) R-squared values in channels C3 and C4 were shown in the left and right panel, respectively, for the three different methods. Statistically significant different R-squared values were found between method G2:CSP/L->LAP/S and G1:LAP/S->CSP/L, between method G2:CSP/L->LAP/S and G3:CSP/S, in channel C3 at the fifth session.

The R-squared value shows the difference of means of EEG mu and beta power between collections of left and right hand motor imagination relative to their band power variance. Thus, it could provide information about electrophysiological changes across training sessions. The group averaged R-squared value over all of the participants for each decoding method was calculated and is visualized in Figure 6. In each row, the group average R-squared value with respect to each method is displayed, and in each column the group averaged results with respect to each session is shown. Note that, for the decoding method of G1:LAP/S and G2:CSP/L, the subjects were switched-over into the other processing and decoding scheme at the fourth session. The color scale for each method is globally normalized to indicate the R-squared values relative to each method. For all of the three rows, focal distributions around channels C3 and C4 show stronger R-squared values, which implies sensorimotor areas are actively modulated by all of the three methods. Specifically, a mixed repeated measures ANOVA was used to assess whether there is a difference of R-squared values between the different methods across sessions in channels C3 and C4. Similar to the online performance analysis, the first three sessions and the last two sessions were analyzed separately. There is a significant main effect of method [*F*_(2, 42)_ = 5.47, *p* = 0.008] and interaction effect [*F*_(2, 42)_ = 3.25, *p* = 0.049] for the R-squared values at channel C3 in the ANOVA accounting for the three groups and final two sessions. The *post-hoc* linear mixed effect models (lme) and Tukey's Test were performed between each method for multiple comparisons of means. The results are summarized in Figure 7. There is a significant difference in R-squared values at channel C3 between methods G2:CSP/L->LAP/S and G1:LAP/S->CSP/L (*p* = 0.0015), between methods G2:CSP/L->LAP/S and G3:CSP/S (*p* = 0.0007) at the fifth session. This indicates that EEG mu and beta waves are more consistently modulated for the LAP/S method at the fifth training session on the group level.

### Typical spatial patterns derived by the CSP/L method

**Figure 8:**
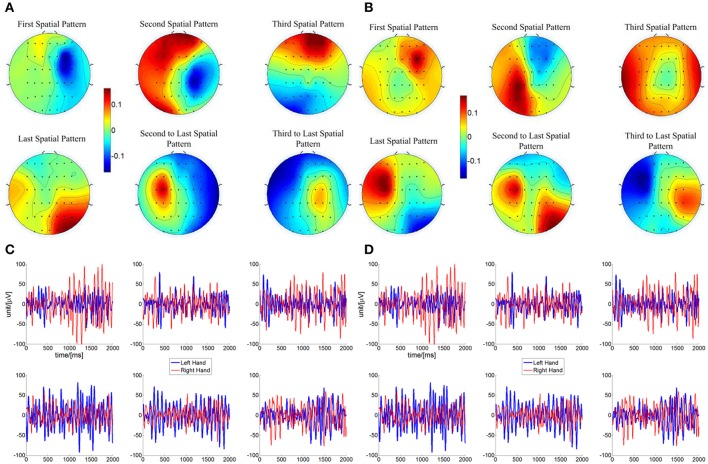
Topography of spatial patterns calculated by CSP/L and corresponding temporal dynamics for a particular subject in the online BCI control. **(A)** Three pairs of spatial patterns corresponding to the three pairs of the highest ranked eigenvalues were used for a particular subject in one run of a session for online BCI control. The first and second spatial pattern in the first row together with the second and third to last spatial patterns in the second row show neurophysiological consistency with the event-related (de)synchronization (ERD/ERS) which is a typical signature of motor imagery tasks. The third spatial pattern and last spatial pattern might capture the non-sensorimotor related activities which happened to be featured by the algorithm. **(B)** Similar spatial patterns in another run of a session for online BCI control, indicating variability in such spatial patterns used for online classification. **(C)** Temporal signals after the spatial filtering from two correct hit trials-left hand imagination and right hand imagination, respectively. The spatial filters were derived from the same CSP transformation which generated the spatial patterns in **(A)**. **(D)** Similar temporal signals after spatial filtering from another two correct hit trials in the run which generate the spatial patterns in **(B)**.

**Figure 9:**
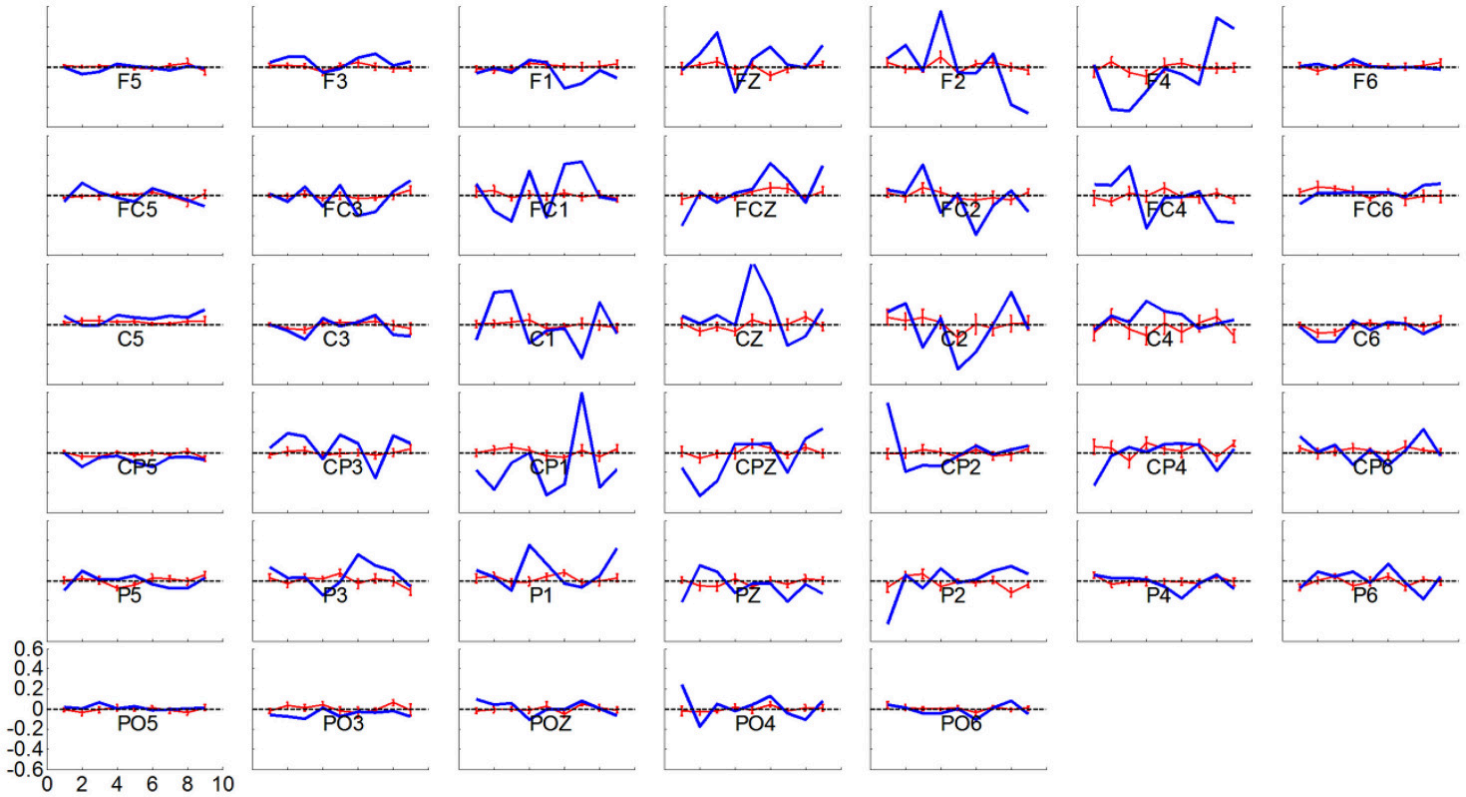
Variation in weighting coefficient for each electrode by the CSP method for a particular subject in blue line and averaged over subjects ± S.E.M in red line, across runs, in a session of online BCI control. The spatial filter for each subject was normalized and then averaged over all of the subjects in a session. A horizontal dash line at y = 0 is shown in each panel.

Considering the results of different online BCI performance shown in the above analysis, feature extraction and classification were analyzed in the current section to probe the EEG for what factors might affect the progress of online BCI performance. Since similar linear classifier and adaptation schemes were used for all of the LAP/S, CSP/L, and CSP/S groups, there is little chance that the classifiers cause the different online BCI performance. We therefore focus on the feature extraction method which is more likely to result in performance differences. An important reason for incorporating multiple channels is the expectation of deriving more task-related information through advanced signal processing algorithms, like spatial filtering. The scalp topographies of three pairs of the highest ranked spatial patterns calculated by CSP/L for each subject and each run during each session were explored with the above question in mind. A typical example for a particular subject is shown in Figure 8. In Figure 8A, spatial patterns derived by CSP/L for one run are shown; the first and second spatial pattern in the first row together with the second and third to last spatial patterns in the second row display neurophysiological consistency with the expected location of event-related (de)synchronization (ERD/ERS) which is a typical signature of motor imagery tasks. Contrasting with these four patterns, the third spatial pattern and last spatial pattern might capture non-sensorimotor related activities of the EEG, such as frontal and occipital activities which happen to be extracted by the algorithm. The temporal dynamics of ERD/ERS filtered by CSP/L, corresponding to the spatial patterns in Figure 8A, are shown in Figure 8C; in general, the temporal signals for right-hand imagination (red line) show larger amplitude oscillations than signals for left-hand imagination (blue line) in the first row of Figure 8C and vice versa in the second row. Similar spatial patterns for another run are shown in Figure 8B; a similar combination that fit with prior neurophysiological knowledge about sensorimotor related activities and non-sensorimotor related activities were observed. The temporal dynamics of ERD/ERS for another two left-hand and right-hand imagery trials are shown in the Figure 8D with their spatial patterns of CSP/L corresponds to Figure 8B. In general, the temporal dynamics of those sensorimotor related activities and non-sensorimotor related activities show similar ERD/ERS at the interested frequency band, but the spatial distributions might vary run by run. An obvious difference between the multichannel or small channel configuration via CSP/L or CSP/S decoding and the small channel configuration via LAP/S decoding was that the weighting coefficient for each electrode (spatial filtering) via CSP/L or CSP/S was automatically derived and adapted by the data. On the contrary, the weight coefficients for the electrodes were fixed throughout the whole sessions of training via LAP/S decoding. The variation of the coefficient for each electrode by CSP method for a particular subject and averaged over subjects is depicted in Figure 9 across runs within a single session in the online BCI control. The spatial filter for each subject was normalized in order to make the average across subjects unbiased. There were clear variations for each coefficient and each subject across each run in a session. The change of coefficient optimized by CSP method caused the change of spatial pattern on a run by run basis which might disturb subjects' consistent modulation of sensorimotor rhythms; this is well reflected as an example in Figure 8 as well.

## Discussion

### BCI performance progress with respect to decoding method and number of channels

The number of channels used in offline analyses has been widely considered to be a critical factor that affects the performance of separating and classifying different motor imagery tasks (Lal et al., 2004; Blankertz et al., 2008b; Sannelli et al., 2010; Arvaneh et al., 2011; Ang et al., 2012; Meng et al., 2013; Shan et al., 2015; Qiu et al., 2016). However, in the current online study, changing the number of channels from 40 electrodes (multichannel CSP/L configuration) to nine electrodes (CSP/S), while maintaining the same decoding method, resulted in no significant difference in group averaged PVC across multiple sessions (refer to Figure 3). Another observation was that there was no trend of improvement in performance across the training sessions for the decoding method of either CSP/L or CSP/S. However, there was a significant improvement of performance from the first session to the third session for the G1:LAP/S compared to G2:CSP/L although a relatively lower initial group averaged performance was observed at the first session. Note that G2:CSP/L showed an overall higher average performance over the three sessions, but there was no significant difference between G1 and G2 from session 2 to session 3. For the last two sessions after switching-over methods, interestingly we found that G2:CSP/L->LAP/S showed significant improvement in change of BCI performance between session 4 and session 5 compared to G1:LAP/S->CSP/L; the performance of the CSP/S group without switching-over presented plateaued performance at the first session and varied a little bit across five sessions. No significant effects were found among the three methods at the end of training session five.

The topographies of the group averaged R-squared values of all three methods in sensor space (Figure 6) clearly show that modulation of brain rhythms near the left C3 and right C4 channels were induced by all methods across the multiple training sessions. This might imply that subjects successfully learned to modulate broad band alpha and beta rhythms from the bilateral motor cortical areas, however, the efficiency of modulation is different at the end of the last session (session 5). G2:CSP/L->LAP/S has a significantly higher group averaged R-squared value compared to the other two groups in channel C3 at session 5. From the topographies of group averaged R-squared values via the LAP/S, the regions of noticeable modulation initially covered a large portion of cortical areas and showed smaller R-squared values around C3 and C4. In later sessions, especially at the fifth session, larger R-squared values were induced in focal regions around the bilateral motor cortical areas. This concept is in line with the findings of the fMRI studies which have compared the cortical activation maps of people deemed to be skilled and unskilled at motor imagination (Guillot et al., 2008). This research involving both motor execution and motor imagery has revealed that cortical activation is more distributed in the unskilled motor imagery group than in the skilled motor imagery group. In the current study, subjects using the LAP/S in the first session were considered to be less skilled than in the later sessions. This could partly explain why the group averaged R-squared sensor space topography at the first session was more spread, weaker compared to their corresponding topography at the latter sessions. However, this trend did not appear in the two CSP decoding groups using either the multichannel configuration or small channel configuration. Contrary to this concept, both of the two CSP decoding groups show plateaued group performance at the first training session. This is likely the case because the larger scalp coverage and optimizing properties of the CSP method allows for a more distributed brain modulation to control the BCI, increasing the ease of control at the beginning of training (Figure 5).

This was not too surprising when considering the group averaged PVC across multiple channels. The performance of these two groups started at a high performance right away but fluctuated throughout the sessions and there was no trend of improvement on performance observed throughout the training processes (Figure 3). This might imply that subjects could not consolidate their skills of consistently modulating the alpha and beta rhythms in the electrodes covering a focal region of the sensorimotor areas. The typical spatial patterns derived by CSP/L (Figure 7) and the variation of resulting weighting coefficients for each electrode (Figure 8), corresponding to the variation of spatial patterns run by run, could help to explain this. Based on the CSP algorithm, the spatial patterns are derived through a data-driven approach and there is no guarantee that each spatial pattern is electrophysiologically relevant to motor imagery modulation, which means that non-sensorimotor related modulation could also contribute to the discrimination of the different motor imagery tasks. By detecting various non-sensorimotor related patterns on a run by run or session by session basis, and when considering the non-stationary behavior of the EEG signal itself, dramatic fluctuations of the weighting coefficient for each electrode is a reasonable outcome even though it is detrimental to inducing stable patterns of modulation for the subjects. Thus, due to the dynamic montage weights when using the CSP algorithm, subjects might not concretely consolidate the skills necessary for controlling a BCI; although the CSP method might provide optimal session-specific performance, it could also lead to inconsistent modulation of subjects' brain rhythms. The careful scrutiny of removing those non-sensorimotor related filters might help to alleviate this problem and needs to be carefully examined by additional experiments in the future investigation.

### Indications from switch-over sessions

The ability of operating a BCI for participants is expected to be generalized from one decoding method to another if they are to use the same underlying neurophysiological mechanism. A better decoding method could potentially help the user be more robust to perturbations; the switch-over design among the first two groups was aimed to test this. The group averaged PVC of the first two groups in the fourth session presented similar performance, but showed different improvement of BCI performance in the fifth session. For the participants of G1:LAP/S->CSP/L, an increased average PVC after switching followed by a decreased average PVC in the subsequent session was observed; for the participants in G2:CSP/L->LAP/S, the average PVC showed a smaller improvement after switching and a big improvement at the fifth session although none of these changes is significant. This difference in trends is supported by the mixed effect ANOVA test results; the results (section Online BCI Performance Results) on the change of BCI performance for the last two sessions compared to the third session before switching methods showed a significant effect of the interaction between method and time. Further *post-hoc* analysis shows that there is a significant difference in change of BCI performance between G1:LAP/S->CSP/L and G2:CSP/L->LAP/S at session 5 compared to session 4. Although there is a significant difference in BCI performance between LAP/S and CSP/S at the fifth session before correcting for multiple comparisons, it is not significant after the correction. The analysis for the first three sessions support the idea that LAP/S allows group one to improve on average during the first three sessions; it seems that LAP/S might also allow group two which starts out with CSP/L and switches over to LAP/S for the last two sessions to increase average BCI performance as well, but more data are needed to support this. On the other hand, there is no clear trend shown for either the CSP/L (group one before switching over and group two after switching over) or CSP/S. Considering these analyses, the trend and R-squared topography, the collective results indicate that LAP/S might help the user be more robust to perturbations. Nevertheless, not only does the method affect the online BCI performance over the course of multiple sessions but other factors such as motivation and mental status might also play a large role (Nijber and Kübler, 2010; Ahn and Jun, 2015). Subjects might lose motivation quickly after multiple sessions of the same paradigm, causing performance variation, and must be considered in long-term BCI training (Kübler et al., 2004).

### Limitations of the study and future work

There are many more channel configurations and associated decoding algorithms other than LAP/S, CSP/L and CSP/S available in the non-invasive EEG based BCI literatures (Blankertz et al., 2006; Lotte et al., 2007). We cannot compare all of them through online experimental validation considering limitation of time and resources. However, the two configurations and decoding methods selected in this study are commonly used and consider both the ends of the spectrum in terms of channel numbers. The small channel configuration includes nine electrodes, the peripheral electrodes surrounding channel C3, C4 used to filter out noise and enhance the signal-to-noise ratio of those two channels. Similar trend to improvement of BCI performance by using only channels C3, C4 was observed as well in previous work (Cassady et al., 2014). For the multichannel configuration, previous offline analyses indicate that the performance increases from using a few channels, saturates, and then decrease after an optimal number of channels (Lal et al., 2004; Sannelli et al., 2010; Arvaneh et al., 2011; Meng et al., 2013; Shan et al., 2015; Qiu et al., 2016). In this study, we choose 40 electrodes for the large channel configuration because most offline analysis shows that performance begins to saturate or decrease by using more than 30–50 electrodes (Arvaneh et al., 2011; Shan et al., 2015; Qiu et al., 2016). There are different kinds of algorithms to extract information from signals of multiple channels. CSP is one state-of-the-art decoding method that aims to maximize the difference of class covariance; weight coefficients for multiple channels are automatically optimized through generalized eigenvalue decomposition. There are also different ways to update the classifier for the CSP method. In this study, we chose to update the CSP weight coefficients and classifier using data collected from a buffering pool of 50 trials after each run, where a similar approach was applied in a previous study (Yao et al., 2014). Since the learning process was the major research question in this study, the CSP weight coefficients and classifier update method might not have been a major factor affecting the learning process since the same approach was applied to all subjects across all of sessions. Other methods adjust the weight coefficients according to different evaluation criterions. Those methods could induce similar problems of the frequent weight variations due to the incorporation of non-sensorimotor related modulation. Thus, we speculate that improving BCI performance of motor imagination could be better induced and consolidated by stable and electrophysiologically meaningful patterns focusing on sensorimotor areas regardless the number of channels.

The current design has only three sessions for G1:LAP/S and G2:CSP/L before switching over and two sessions for G1:LAP/S->CSP/L and G2:CSP/L->LAP/S after switching. Since we did not collect longitudinal data, it is difficult to conclude whether LAP/S will ultimately outperform CSP/L even though LAP/S provides more room for learning at the initial session It would be desirable to have more sessions both before and after switching over to better highlight performance trends, if there are any. Also there are variations for inter-session intervals among the subjects due to practical limitation, even though attempts were made to schedule as soon as possible the next session. For human study in a large group (45 subjects) with multiple sessions each, it is practically impossible to optimize all parameters such as number of sessions, inter-session intervals, etc. The motivation for the study and schedule conflicts of subjects might have to be kept in mind before planning longer sessions. However, the present results provide data on important issues that may impact BCI performance. It would also be interesting to see when the subjects' performance would plateau for the LAP/S method in a group level since a significant improvement of BCI performance was found across the first three sessions; this will be our future investigation.

## Conclusion

In this study, two channel configurations representing a small number and large number of channels, along with LAP/S decoding and multichannel decoding algorithm—CSP—were utilized and compared to study the online learning process during multiple sessions. Throughout the multiple learning sessions we found that CSP decoding method, for online BCI control based on the either multiple channels or a small number of channels, shows no difference for BCI online performance but shows high group average performance at the initial sessions than the LAP/S method. Nevertheless, the high performance plateaued at the early training phase and may partially be caused by non-sensorimotor related modulation. Thus, no improvement during multiple sessions was observed. On the contrary, the LAP/S decoding method, for online BCI control via a small numbers of channels, started at a lower group average accuracy, but a trend of improvement and stable pattern of modulation over multiple sessions was observed. The results of the switch-over study imply that LAP/S might help subjects to be resistant to perturbations to a certain degree. These results altogether implicate that devising a successful decoding algorithm for online application specifically requires incorporating consideration of subjects' engagement and learning progress in longitudinal BCI control. With respect to long term BCI use, it appears necessary to exclude non-sensorimotor related modulation, which could facilitate higher performance in single individual sessions but might not be beneficial for skill consolidation in longitudinal sessions.

## Author contributions

JM and BH conceived the project, designed the experiments, interpreted the results and wrote the paper. JM, JO, GJ, SZ, and AB performed the experiments and analyzed the data. BE contributed to data analysis, interpretation of results and the writing of the manuscript.

### Conflict of interest statement

The authors declare that the research was conducted in the absence of any commercial or financial relationships that could be construed as a potential conflict of interest.

## References

[B1] Ahn M. Jun S. C. (2015). Performance variation in motor imagery brain–computer interface: a brief review. J. Neurosci. Methods 243, 103–110. 10.1016/j.jneumeth.2015.01.033 25668430

[B2] Ang K. K. Chin Z. Y. Wang C. Guan C. Zhang H. (2012). Filter bank common spatial pattern algorithm on BCI competition IV datasets 2a and 2b. Front. Neurosci. 6:39. 10.3389/fnins.2012.00039 22479236 PMC3314883

[B3] Arvaneh M. Guan C. Ang K. K. Quek C. (2011). Optimizing the channel selection and classification accuracy in EEG-based BCI. Biomed. Eng. IEEE Trans. 58, 1865–1873. 10.1109/TBME.2011.2131142 21427014

[B4] Baxter B. S. Edelman B. J. Nesbitt N. He B. (2016). Sensorimotor rhythm BCI with simultaneous high definition-transcranial direct current stimulation alters task performance. Brain Stimul. 9, 834–841. 10.1016/j.brs.2016.07.003 27522166 PMC5143161

[B5] Birbaumer N. Hinterberger T. Kubler A. Neumann N. (2003). The thought-translation device (TTD): neurobehavioral mechanisms and clinical outcome. IEEE Trans. Neural Syst. Rehabil. Eng. 11, 120–123. 10.1109/TNSRE.2003.814439 12899251

[B6] Blankertz B. Losch F. Krauledat M. Dornhege G. Curio G. Müller K. R. (2008a). The Berlin Brain–Computer Interface: accurate performance from first-session in BCI-naive subjects. IEEE trans. Biomed. Eng. 55, 2452–2462. 10.1109/TBME.2008.923152 18838371

[B7] Blankertz B. Muller K. R. Krusienski D. J. Schalk G. Wolpaw J. R. Schlogl A.. (2006). The BCI competition III: Validating alternative approaches to actual BCI problems. IEEE Trans. Neural Syst. Rehabil. Eng. 14, 153–159. 10.1109/TNSRE.2006.875642 16792282

[B8] Blankertz B. Tomioka R. Lemm S. Kawanabe M. Müller K. R. (2008b). Optimizing spatial filters for robust EEG single-trial analysis. Signal Process. Mag. IEEE 25, 41–56. 10.1109/MSP.2008.4408441

[B9] Blokland Y. Spyrou L. Lerou J. Mourisse J. Scheffer G. J. van Geffen G.-J.. (2015). Detection of attempted movement from the EEG during neuromuscular block: proof of principle study in awake volunteers. Sci. Rep. 5:12815. 10.1038/srep12815 26248679 PMC4528221

[B10] Carlson T. Millan J. D. R. (2013). “Brain-controlled wheelchairs: a robotic architecture, in IEEE Robotics and Automation Magazine, 20(EPFL-ARTICLE-181698) 65–73. 10.1109/MRA.2012.2229936

[B11] Cassady K. You A. Doud A. He B. (2014). The impact of mind-body awareness training on the early learning of a brain-computer interface. Technology 2, 254–260. 10.1142/S233954781450023X 26086029 PMC4465229

[B12] Chen X. Wang Y. Nakanishi M. Gao X. Jung T. P. Gao S. (2015). High-speed spelling with a noninvasive brain–computer interface. Proc. Natl. Acad. Sci. U.S.A. 112, E6058–E6067. 10.1073/pnas.1508080112 26483479 PMC4640776

[B13] Delorme A. Makeig S. (2004). EEGLAB: an open source toolbox for analysis of single-trial EEG dynamics including independent component analysis. J. Neurosci. Methods 134, 9–21. 10.1016/j.jneumeth.2003.10.009 15102499

[B14] Donati A. R. C. Shokur S. Morya E. Campos D. S. Moioli R. C. Gitti C. M.. (2016). Long-Term training with a brain-machine interface-based gait protocol induces partial neurological recovery in paraplegic patients. Sci. Rep. 6:30383. 10.1038/srep30383 27513629 PMC4980986

[B15] Edelman B. J. Baxter B. He B. (2016). EEG source imaging enhances the decoding of complex right-hand motor imagery tasks. Biomed. Eng IEEE Trans. 63, 4–14. 10.1109/TBME.2015.2467312 26276986 PMC4716869

[B16] Nijber F. Birbaumer N. Kübler A. (2010). The influence of psychological state and motivation on brain–computer interface performance in patients with amyotrophic lateral sclerosis–a longitudinal study. Front. Neurosci. 4:55 10.3389/fnins.2010.00055 20700521 PMC2916671

[B17] Guger C. Ramoser H. Pfurtscheller G. (2000). Real-time EEG analysis with subject-specific spatial patterns for a brain-computer interface (BCI). IEEE Transac. Rehabil. Eng. 8, 447–456. 10.1109/86.895947 11204035

[B18] Guillot A. Collet C. Nguyen V. A. Malouin F. Richards C. Doyon J. (2008). Functional neuroanatomical networks associated with expertise in motor imagery. NeuroImage 41, 1471–1483. 10.1016/j.neuroimage.2008.03.042 18479943

[B19] Hammer E. M. Halder S. Blankertz B. Sannelli C. Dickhaus T. Kleih S.. (2012). Psychological predictors of SMR-BCI performance. Biol. Psychol. 89, 80–86. 10.1016/j.biopsycho.2011.09.006 21964375

[B20] He B. Gao S. Yuan H. Wolpaw J. R. (2013). Brain–computer interfaces, in Neural Engineering, 2nd Edn., ed He B. (Heidelberg; Dordrecht; London; New York, NY: Springer), 87–151.

[B21] Hjorth B. (1975). An on-line transformation of EEG scalp potentials into orthogonal source derivations. Electroencephalogr. Clin. Neurophysiol. 39, 526–530. 10.1016/0013-4694(75)90056-5 52448

[B22] Jin J. Allison B. Z. Brunner C. Wang B. Wang X. Zhang J.. (2010). P300 Chinese input system based on Bayesian, LDA. Biomed. Tech. Biomed. Eng. 55, 5–18. 10.1515/BMT.2010.003 20128741

[B23] Jin J. Allison B. Z. Sellers E. W. Brunner C. Horki P. Wang X.. (2011). An adaptive P300-based control system. J. Neural Eng. 8:036006. 10.1088/1741-2560/8/3/036006 21474877 PMC4429775

[B24] Kaiser V. Bauernfeind G. Kreilinger A. Kaufmann T. Kubler A. Neuper C.. (2014). Cortical effects of user training in a motor imagery based brain–computer interface measured by fNIRS and EEG. NeuroImage 85, 432–444. 10.1016/j.neuroimage.2013.04.097 23651839

[B25] King C. E. Wang P. T. McCrimmon C. M. Chou C. C. Do A. H. Nenadic Z. (2015). The feasibility of a brain-computer interface functional electrical stimulation system for the restoration of overground walking after paraplegia. J. Neuroeng. Rehabil. 12:80. 10.1186/s12984-015-0068-7 26400061 PMC4581411

[B26] Kübler A. Neumann N. Wilhelm B. Hinterberger T. Birbaumer N. (2004). Predictability of brain-computer communication. J. Psychophysiol. 18, 121–129. 10.1027/0269-8803.18.23.121

[B27] LaFleur K. Cassady K. Doud A. Shades K. Rogin E. He B. (2013). Quadcopter control in three-dimensional space using a noninvasive motor imagery-based brain–computer interface. J. Neural Eng. 10:046003. 10.1088/1741-2560/10/4/046003 23735712 PMC3839680

[B28] Lal T. N. Schröder M. Hinterberger T. Weston J. Bogdan M. Birbaumer N.. (2004). Support vector channel selection in BCI. Biomed. Eng. IEEE Trans. 51, 1003–1010. 10.1109/TBME.2004.827827 15188871

[B29] Lawson J. (2014). Chapter 9: Design and Analysis of Experiments with R. (London: CRC Press), 351–357.

[B30] Lotte F. Congedo M. Lécuyer A. Lamarche F. Arnaldi B. (2007). A review of classification algorithms for EEG-based brain–computer interfaces. J. Neural Eng. 4, R1–R13. 10.1088/1741-2560/4/2/R01 17409472

[B31] Lotte F. Guan C. (2011). Regularizing common spatial patterns to improve BCI designs: unified theory and new algorithms. IEEE Trans. Biomed. Eng. 58, 355–362. 10.1109/TBME.2010.2082539 20889426

[B32] McFarland D. J. (2015). The advantages of the surface Laplacian in brain–computer interface research. Int. J. Psychophysiol. 97, 271–276. 10.1016/j.ijpsycho.2014.07.009 25091286 PMC4312749

[B33] McFarland D. J. McCane L. M. David S. V. Wolpaw J. R. (1997). Spatial filter selection for EEG-based communication. Electroencephalogr. Clin. Neurophysiol. 103, 386–394. 10.1016/S0013-4694(97)00022-2 9305287

[B34] Meng J. Huang G. Zhang D. Zhu X. (2013). Optimizing spatial spectral patterns jointly with channel configuration for brain–computer interface. Neurocomputing 104, 115–126. 10.1016/j.neucom.2012.11.004

[B35] Meng J. Sheng X. Zhang D. Zhu X. (2014). Improved semisupervised adaptation for a small training dataset in the brain–computer interface. IEEE J. Biomed. Health Inform. 18, 1461–1472. 10.1109/JBHI.2013.2285232 24122610

[B36] Meng J. Zhang S. Bekyo A. Olsoe J. Baxter B. He B. (2016). Noninvasive Electroencephalogram based control of a robotic arm for reach and grasp tasks. Sci. Rep. 6:38565. 10.1038/srep38565 27966546 PMC5155290

[B37] Neuper C. Scherer R. Reiner M. Pfurtscheller G. (2005). Imagery of motor actions: differential effects of kinesthetic and visual–motor mode of imagery in single-trial EEG. Cogn. Brain Res. 25, 668–677. 10.1016/j.cogbrainres.2005.08.014 16236487

[B38] Pfurtscheller G. Brunner C. Schlögl A. Da Silva F. L. (2006). Mu rhythm (de) synchronization and EEG single-trial classification of different motor imagery tasks. NeuroImage 31, 153–159. 10.1016/j.neuroimage.2005.12.003 16443377

[B39] Pfurtscheller G. Müller G. R. Pfurtscheller J. Gerner H. J. Rupp R. (2003). ‘Thought’–control of functional electrical stimulation to restore hand grasp in a patient with tetraplegia. Neurosci. Lett. 351, 33–36. 10.1016/S0304-3940(03)00947-9 14550907

[B40] Pichiorri F. De Vico Fallani F. Cincotti F. Babiloni F. Molinari M. Kleih S.. (2011). Sensorimotor rhythm-based brain–computer interface training: the impact on motor cortical responsiveness. J. Neural Eng. 8:025020. 10.1088/1741-2560/8/2/025020 21436514

[B41] Pichiorri F. Morone G. Petti M. Toppi J. Pisotta I. Molinari M.. (2015). Brain–computer interface boosts motor imagery practice during stroke recovery. Ann. Neurol. 77, 851–865. 10.1002/ana.24390 25712802

[B42] Qin J. Li Y. Sun W. (2007). A semisupervised support vector machines algorithm for BCI systems. Comput. Intell. Neurosci. 2007, 1–9. 10.1155/2007/94397 PMC2267906 18368141

[B43] Qiu Z. Allison B. Z. Jin J. Zhang Y. Wang X. Li W.. (2017). Optimized motor imagery paradigm based on imagining Chinese characters writing movement. IEEE Trans. Neural Syst. Rehabil. Eng. 25, 1009–1017. 10.1109/TNSRE.2017.2655542 28113345

[B44] Qiu Z. Jin J. Lam H. K. Zhang Y. Wang X. Cichocki A. (2016). Improved SFFS method for channel selection in motor imagery based BCI. Neurocomputing 207, 519–527. 10.1016/j.neucom.2016.05.035

[B45] Ramos-Murguialday A. Broetz D. Rea M. Läer L. Yilmaz Ö. Brasil F. L.. (2013). Brain–machine interface in chronic stroke rehabilitation: a controlled study. Ann. Neurol. 74, 100–108. 10.1002/ana.23879 23494615 PMC3700597

[B46] Ramoser H. Muller-Gerking J. B. Pfurtscheller G. (2000). Optimal spatial filtering of single trial EEG during imagined hand movement. IEEE Trans. Rehabil. Eng. 8, 441–446. 10.1109/86.895946 11204034

[B47] Royer A. S. Doud A. J. Rose M. L. He B. (2010). EEG control of a virtual helicopter in 3-dimensional space using intelligent control strategies. Neural Syst. Rehabil. Eng. IEEE Trans. 18, 581–589. 10.1109/TNSRE.2010.2077654 20876032 PMC3037732

[B48] Samek W. Kawanabe M. Müller K. R. (2014). Divergence-based framework for common spatial patterns algorithms. IEEE Rev. Biomed. Eng. 7, 50–72. 10.1109/RBME.2013.2290621 24240027

[B49] Sannelli C. Dickhaus T. Halder S. Hammer E. M. Müller K. R. Blankertz B. (2010). On optimal channel configurations for SMR-based brain–computer interfaces. Brain Topogr. 23, 186–193. 10.1007/s10548-010-0135-0 20162347

[B50] Schalk G. McFarland D. J. Hinterberger T. Birbaumer N. Wolpaw J. R. (2004). BCI2000: a general-purpose brain-computer interface (BCI) system. Biomed. Eng. IEEE Trans. 51, 1034–1043. 10.1109/TBME.2004.827072 15188875

[B51] Shan H. Xu H. Zhu S. He B. (2015). A novel channel selection method for optimal classification in different motor imagery BCI paradigms. Biomed. Eng. Online 14:93. 10.1186/s12938-015-0087-4 26489759 PMC4618360

[B52] Tangermann M. Müller K. R. Aertsen A. Birbaumer N. Braun C. Brunner C.. (2012). Review of the BCI competition IV. Front. Neurosci. 6:55. 10.3389/fnins.2012.00055 22811657 PMC3396284

[B53] Wolpaw J. R. Birbaumer N. McFarland D. J. Pfurtscheller G. Vaughan T. M. (2002). Brain–computer interfaces for communication and control. Clin. Neurophysiol. 113, 767–791. 10.1016/S1388-2457(02)00057-3 12048038

[B54] Wolpaw J. R. McFarland D. J. (2004). Control of a two-dimensional movement signal by a noninvasive brain-computer interface in humans. Proc. Natl. Acad. Sci. U.S.A. 101, 17849–17854. 10.1073/pnas.0403504101 15585584 PMC535103

[B55] Yao L. Meng J. Sheng X. Zhang D. Zhu X. (2014). A novel calibration and task guidance framework for motor imagery BCI via a tendon vibration induced sensation with kinesthesia illusion. J. Neural Eng. 12:016005. 10.1088/1741-2560/12/1/016005 25461477

[B56] Zich C. Debener S. Kranczioch C. Bleichner M. G. Gutberlet I. De Vos M. (2015). Real-time EEG feedback during simultaneous EEG–fMRI identifies the cortical signature of motor imagery. NeuroImage 114 438–447. 10.1016/j.neuroimage.2015.04.020 25887263

